# CUT Domain Proteins in DNA Repair and Cancer

**DOI:** 10.3390/cancers13122953

**Published:** 2021-06-12

**Authors:** Zubaidah M. Ramdzan, Elise Vickridge, Camila C. F. Faraco, Alain Nepveu

**Affiliations:** 1 Goodman Cancer Research Centre, McGill University, 1160 Pine Avenue West, Montreal, QC H3A 1A3, Canada; zubaidah.mohamedramdzan@mcgill.ca (Z.M.R.); elise.vickridge@mail.mcgill.ca (E.V.); camila.fragafaraco@mail.mcgill.ca (C.C.F.F.); 2 Departments of Biochemistry, McGill University, 1160 Pine Avenue West, Montreal, QC H3A 1A3, Canada; 3 Departments of Medicine, McGill University, 1160 Pine Avenue West, Montreal, QC H3A 1A3, Canada; 4 Departments of Oncology, McGill University, 1160 Pine Avenue West, Montreal, QC H3A 1A3, Canada

**Keywords:** CUX1, CUX2, SATB1, CUT domains, base excision repair, RAS, reactive oxygen species

## Abstract

**Simple Summary:**

Genetic integrity is ensured by complex groups of proteins involved in DNA repair. In particular, base damage is repaired by enzymes of the base excision repair pathway. Recent studies have revealed that some transcription factors can function as accessory factors that stimulate the enzymatic activities of these DNA repair enzymes. It is well known that defects in DNA repair mechanisms cause the accumulation of changes in DNA, called mutations, that increase the possibility that cells become tumorigenic. Paradoxically, once they have emerged certain cancer cells are acutely dependent on the heightened activities of base excision repair enzymes because their metabolism generates highly reactive molecules that cause multiple types of damage to bases. In this context, the function of accessory factors becomes essential to cancer cell survival. As a by-product of this adaptation, cancer cells become more resistant to therapies that cause DNA damage, such as chemotherapy and radiation.

**Abstract:**

Recent studies revealed that CUT domains function as accessory factors that accelerate DNA repair by stimulating the enzymatic activities of the base excision repair enzymes OGG1, APE1, and DNA pol β. Strikingly, the role of CUT domain proteins in DNA repair is exploited by cancer cells to facilitate their survival. Cancer cells in which the RAS pathway is activated produce an excess of reactive oxygen species (ROS) which, if not counterbalanced by increased production of antioxidants, causes sustained oxidative DNA damage and, ultimately, cell senescence. These cancer cells can adapt by increasing their capacity to repair oxidative DNA damage in part through elevated expression of CUT domain proteins such as CUX1, CUX2, or SATB1. In particular, CUX1 overexpression was shown to cooperate with RAS in the formation of mammary and lung tumors in mice. Conversely, knockdown of *CUX1*, *CUX2*, or *SATB1* was found to be synthetic lethal in cancer cells exhibiting high ROS levels as a consequence of activating mutations in *KRAS*, *HRAS*, *BRAF*, or *EGFR*. Importantly, as a byproduct of their adaptation, cancer cells that overexpress CUT domain proteins exhibit increased resistance to genotoxic treatments such as ionizing radiation, temozolomide, and cisplatin.

## 1. Introduction

### 1.1. CUT Domain Proteins

**Figure 1:**
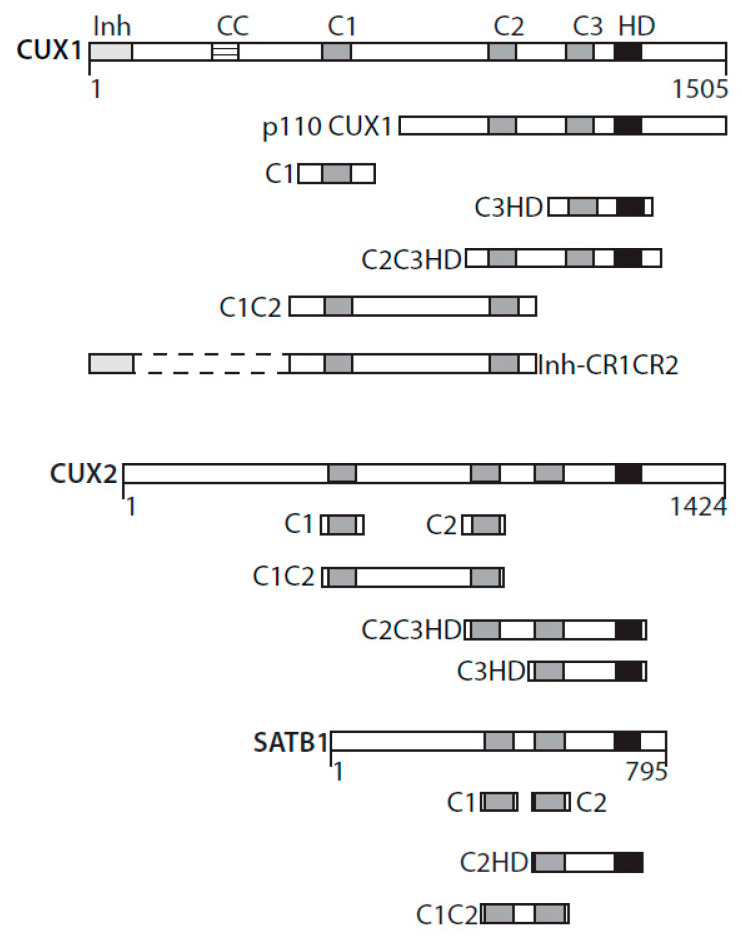
CUT domains active in base excision repair. Diagrammatic representation of CUX1, CUX2, and SATB1 and the recombinant CUT domain proteins active in base excision repair. Inh, DNA-binding inhibitory domain; CC, coiled-coil; C1, C2, C3, CUT domains 1, 2, and 3; HD, homeodomain.

The term “cut” derives from the truncated or cut wing phenotype observed in a *cut* mutant of *Drosophila melanogaster* [1]. CUT domains are regions of approximately 70 amino acids present in one, two, or three copies in proteins encoded by the CUT superclass homeobox genes. Three CUT domains, also referred to as Cut repeats, are present in CUX1 and CUX2 (CUT homeobox) proteins (Figure 1) [2,3]. Two CUT domains are found in SATB1 and SATB2 proteins (Figure 1) [4]. The ONECUT class genes code for proteins with only a single CUT domain [5]. Proteins of these three classes also contain a CUT-type homeodomain [2,3,4,5]. CUT domains were originally characterized as DNA-binding domains [6,7,8]. Only recently were CUT domains shown to be involved in protein–protein interactions [9,10,11].

A single CUT domain cannot bind to DNA but can do so in conjunction with another CUT domain or with the homeodomain within the same protein [12]. For example, experiments with recombinant proteins revealed that CUT domain 1 (C1) of the CUX1 protein can bind DNA together with CUT domain 2 (C2) or with the homeodomain (HD) (Figure 1) [12]. Interestingly, the C1C2 and C1HD proteins exhibit strikingly different DNA-binding kinetics: C1C2 binds to DNA with very fast “on” and “off” rates, whereas any recombinant protein that includes a homeodomain in addition to a CUT domain binds DNA more slowly but more stably [12]. Intriguingly, the full-length CUX1 protein binds to DNA with similar DNA-binding specificity and kinetics to the C1C2 recombinant protein [12].

For a description of the role of CUT domain proteins in transcriptional regulation, we refer the readers to previous reviews [13,14,15]. Pertinent to the subject of this review, it should be noted that the p110 CUX1 protein was shown to transcriptionally activate several genes involved in the DNA damage response [16] and the spindle assembly checkpoint [17]. These results indicate that CUX1 regulates a transcriptional program that is necessary to mount an efficient response to DNA damage and ensure proper chromosome segregation.

### 1.2. The Need for CUX1 in RAS-Induced Tumor Formation

**Figure 2:**
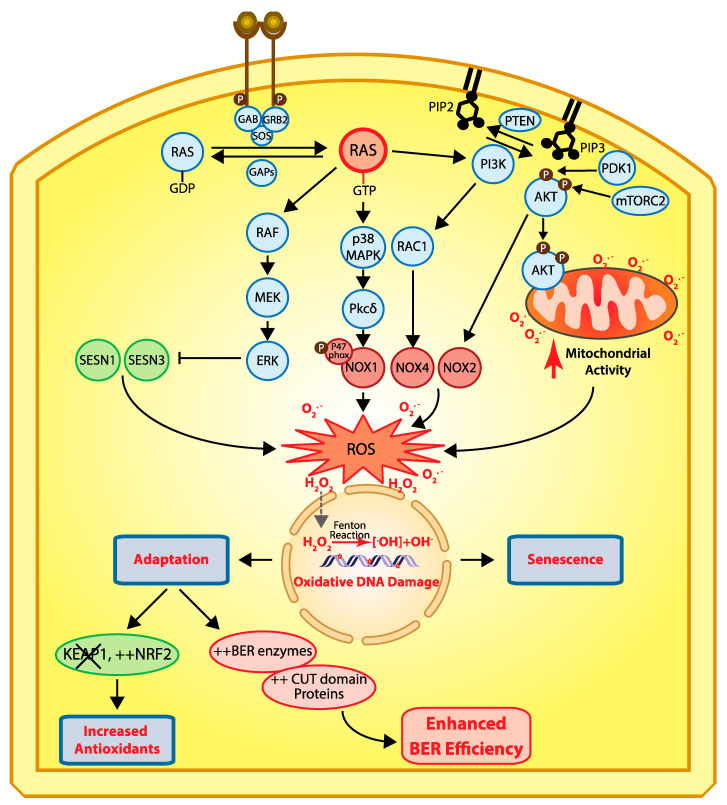
Cellular responses to RAS-driven production of reactive oxygen species. In physiological conditions, RAS proteins (KRAS, HRAS, and NRAS) alternate between their GDP- and GTP-bound states which are regulated by guanine nucleotide exchange factors (GEFs) and GTPase-activating proteins (GAPs) [40]. Binding of a receptor tyrosine kinase (RTK) to its ligand induces its dimerization and autophosphorylation, which in turn allows the recruitment of SH2 domain-containing proteins such as GRB2 that recruits the GEF protein SOS which then activates RAS [41,42]. In cancer, RAS is activated by a number of mechanisms including activating mutations in one of the RAS genes, overexpression of RTKs, and loss of GAP proteins (reviewed in [43]). In its GTP-bound state, RAS activates a number of downstream effector pathways that elevate the level of reactive oxygen species (ROS) in multiple ways [23]. The mitogen-activated protein kinase (MAPK) pathway leads to the transcriptional activation of NOX1 which codes for NADPH oxidase 1, a member of the NADPH oxidase enzyme family that catalyzes the one-electron transfer of oxygen to generate superoxide at the plasma membrane [21,44]. In addition, phosphorylation of the p47phox Nox1 subunit by protein kinase C δ (PKCδ) induces its translocation to the plasma membrane [45]. Another study provided evidence that RAS-induced ROS production is dependent on RAC1 and NOX4, another member of the NADPH oxidase enzyme family [46]. The RAF–MEK–ERK pathway causes the transcriptional repression of sestrin family genes, SESN1 and SESN3, which code for antioxidant modulators of peroxiredoxins [47]. Another important signaling pathway downstream of RAS is the pleiotropic PI3K/AKT pathway [43]. Importantly, the PI3K/AKT pathway is frequently activated in human cancers in a RAS-independent manner following a mutation or amplification of PIK3CA which codes for the p110α catalytic subunit of the phosphatidylinositol 3-kinase (PI3K) or following inactivation of PTEN, a gene encoding the tumor suppressor phosphatase and tensing homolog, which dephosphorylates phosphatidylinositol 3,4,5-triphosphate (PIP_3_) to phosphatidylinositol 4,5-bisphosphate (PIP_2_), thereby terminating PI3K-dependent signaling [43]. The accumulation of PIP3 facilitates the localization of PH domain-containing proteins such as AKT and PDK1 to the plasma membrane where AKT is activated following its phosphorylation by PDK1 [48,49,50] and mTORC2 [51,52,53]. Activated AKT phosphorylates a large number of proteins involved in diverse cellular processes [43]. Importantly, PI3K/AKT signaling has been implicated in the activation of NOX activity [54,55]. Moreover, phosphorylation of AKT induces its translocation to the mitochondrial matrix and inner membrane [56], where it can phosphorylate GSK-3β, thereby lifting the negative regulation of pyruvate dehydrogenase and α-ketoglutarate dehydrogenase, two complexes that produce superoxide and H_2_O_2_ [57,58,59]. Note that reactive oxygen species (ROS) do not travel through the cell since they react with the next molecule. However, ROS can be converted into H_2_O_2_ which moves into the cells and penetrates the nucleus, where it can be converted into hydroxyl radicals (^•^OH^−^) when it comes in contact with ferrous ions [60]. Hydroxyl radicals in turn cause oxidative DNA damage, and sustained DNA damage eventually causes cellular senescence. Cells can adapt via two mechanisms: (1) increased expression of antioxidants following inactivation of KEAP1 and/or upregulation of NRF2 [35], or (2) increased expression of BER enzymes and accessory factors such as CUT domains [9,10,11,38,61,62].

A role for CUX1 in DNA repair was first suggested from the analysis of mammary tumors that develop in MMTV-CUX1 transgenic mice [9]. Accordingly, 44% of tumors from these transgenic mice exhibited a spontaneous missense mutation at codon 12 or 61 of *Kras* that resulted in the activation of this oncogene [9]. The prevalence of these mutations indicates that spontaneous activating mutations within *Kras* occur frequently; however, we rarely observe tumors arising from such mutations in the wild-type mouse. This suggests that other cooperating events are needed for tumor development following activation of a *RAS* gene. The cooperation between CUX1 and Kras was confirmed using lentiviral infections in the lung [9]. Mice that received a CUX1-expressing lentivirus in addition to the Kras lentivirus developed a higher number of tumors than mice infected only with the Kras lentivirus [9]. These tumors were of larger size and progressed further along the pathological spectrum. While the *KRAS^G12D^* mice developed solely grade 1 adenomas, mice expressing KRAS^G12D^ and CUX1 developed higher-grade adenomas and one large adenocarcinoma [9]. That ectopic expression of KRAS^G12D^ alone produced only low-grade adenomas was not unexpected. Earlier studies showed that *RAS* oncogenes cannot transform primary cells [18]. Cells that harbor an activated RAS oncogene produce an excess of reactive oxygen species (ROS) that cause oxidative DNA damage and, ultimately, cellular senescence (Figure 2) [19,20,21]. Cellular senescence can also be observed in cells that exhibit constitutive activation of the RAS pathway as a consequence of a mutation in another gene of the same signaling pathway (reviewed in [22,23]). This has been documented not only in tissue culture and mouse models [24,25,26], but also in premalignant human colon adenomas [27,28,29], as well as in human benign lesions caused by the *BRAF^V600E^* mutation [30] or NF1 inactivation [31]. In this context, cellular senescence has been deemed a tumor suppression mechanism [32]. Unfortunately, cancer cells can sometimes adapt and continue to proliferate despite producing high levels of ROS. Cancer cells can reduce ROS levels by increasing the expression of antioxidants [33,34,35], notably following inactivation of the *KEAP1* tumor suppressor gene, an event observed in 15–30% of cancers [36]. Alternatively, what is becoming increasingly evident is that cancer cells can adapt to elevated ROS by increasing their capacity to repair oxidative DNA damage [9,37,38]. This can be achieved through increased expression of enzymes of the base excision repair (BER) pathway, as well as BER accessory factors [38]. Indeed, ectopic expression of CUX1 together with an activated RAS oncogene did not affect ROS levels, but greatly reduced genomic DNA damage, as well as the proportion of senescent cells in the population [9]. Further experiments showed that CUX1 accelerated the repair of oxidative DNA damage caused by exposure to H_2_O_2_ [9,39]. In particular, single-cell gel electrophoresis (comet assay) performed at pH 10 after treatment with the formamidopyrimidine DNA glycosylase (FPG) enzyme revealed that CUX1 accelerates the repair of oxidized purines [9,39]. In turn, CUX1 knockdown delays the repair of oxidized purines [9]. These results were confirmed by measuring the levels of 8-oxodeoxyguanines in genomic DNA [9].

Subsequent experiments established that *CUX1* knockdown is synthetic lethal in all cancer cells exhibiting high levels of ROS due to an activating mutation in a *RAS* gene (Hs578T*^HRAS^*, MDA-MB-231*^KRAS^*, DLD-1*^KRAS^*, HCT116*^KRAS^*, KE37*^NRAS^*), another gene in the pathway (HT29*^BRAF^*), or an upstream receptor tyrosine kinase (HCC827*^EGFR^*) ([9,38] and Ramdzan et al., in preparation). In contrast, CUX1 knockdown did not reduce the clonogenic efficiency of cell lines that exhibit relatively low ROS levels [38]. The case of the A549 cells is particularly enlightening. These cells carry an activating mutation in the *KRAS* oncogene but still exhibit low ROS, because inactivation of the *KEAP1* tumor suppressor gene in these cells leads to greater accumulation of NRF2 in the nucleus and increased activation of genes coding for antioxidants [36].

## 2. Structure/Function Analysis Implicate the CUT Domains in DNA Repair

The increase in 8-oxodeoxyguanines (8-oxoG) detected in genomic DNA following *CUX1* knockdown led us to verify the expression and activity of the 8-oxodeoxyguanine DNA glycosylase, OGG1. While OGG1 steady-state levels remained unchanged, cleavage of oligonucleotides containing an 8-oxoG base was drastically reduced in whole-cell extracts from CUX1 knockdown cells [9,39]. Coimmunoprecipitation assays provided evidence that OGG1 and CUX1 can be part of the same complex in cells, whereas pulldown assays indicated that the two proteins can directly interact with each other [9,39]. The 8-oxoG cleavage assay was, therefore, performed with the purified OGG1 enzyme in the presence of His-tagged recombinant proteins containing various regions of the CUX1 protein. These assays revealed that any protein containing at least one CUT domain was able to stimulate the enzymatic activities of OGG1. Recombinant CUX1 proteins able to stimulate OGG1’s enzymatic activities included CUT domain 1 (C1), CUT domains 1 and 2 (C1C2), CUT domains 2 and 3 plus the CUT homeodomain (C2C3HD), and CUT domain 3 plus the CUT homeodomain (C3HD) (Figure 1) [9,39]. In contrast, the CUT homeodomain expressed on its own or the carboxy-terminal domain, in which transcriptional repression domains were previously identified, were inactive in these assays [39]. Similarly, all other tested transcription factors or DNA-binding domains were inactive, including ERRα, ERRα DNA-binding domain (ERRα-DBD), TCF-DBD, PPARδ, HoxB3, and Gal4 [39]. Subsequent experiments with recombinant proteins derived from CUX2, a protein whose expression is restricted to neuronal cells of the central and peripheral nervous system, produced similar results [10]. In the case of SATB1, which contains only two CUT domains, OGG1 stimulation was observed with C1, C2, C1C2, and C2HD proteins [11] (Figure 1).

In mammalian cells, ectopic expression of a recombinant protein containing only the CUT domains 1 and 2 fused to a nuclear localization signal (C1C2-NLS) (to target it to the nucleus in the absence of the Cut homeodomain) was sufficient to accelerate the repair of oxidative DNA damage after treatment with H_2_O_2_ [9,39]. Importantly, we demonstrated that this recombinant protein, C1C2-NLS, is devoid of transcription activation potential [9,39]. Gene expression analysis confirmed that known transcriptional targets of the p110 CUX1 factor, as well as genes of the base excision repair pathway, were not upregulated by C1C2-NLS [9,39]. 

The p110 CUX1 protein isoform is produced by proteolytic processing of CUX1 in the late G1 phase of the cell cycle [63,64]. In contrast to the full-length CUX1 protein which exhibits very fast DNA-binding kinetics, the p110 CUX1 isoform binds stably to DNA and is able to function as a transcriptional activator or repressor depending on promoter context [12]. Despite the fact that the p110 CUX1 isoform represents at most 5% of all CUX1 proteins in the cells, most confirmed transcriptional targets of CUX1 are in fact regulated by p110 CUX1 [16,17,65,66,67,68,69,70]. Ectopic expression of p110 CUX1 was able to accelerate DNA repair following treatment with H_2_O_2_; however, we believe that the endogenous p110 CUX1 protein is not abundant enough in cells to have much impact on DNA repair [9].

## 3. Stimulation of OGG1—Mechanism of Action

OGG1, like other DNA glycosylases specific for oxidized bases, is endowed with two enzymatic functions: a glycosylase activity that removes the oxidized purine and an apurinic (AP)-lyase activity that introduces a single-strand break [71,72]. When observing a greater amount of the cleaved single-strand in the 8-oxoG cleavage assay, it is not possible to distinguish which reaction was stimulated or whether both were stimulated. However, incubation of the reaction products with NaOH prior to gel electrophoresis produces a single-strand break at all abasic sites and, therefore, enables one to monitor specifically the glycosylase activity [73]. These assays revealed that CUT domains stimulate the glycosylase activity of OGG1 [10,39]. In addition, AP-lyase reaction proceeds through the formation of a Schiff-base intermediate, whereby the enzyme is covalently linked to the DNA sugar backbone concomitantly with the removal of the base [71,73]. Addition of sodium borohydride to trap this intermediate revealed that CUT domains stimulate Schiff-base formation by OGG1 [11,39]. These findings led us to investigate the very first step in the reaction: recognition of 8-oxodeoxyguanine by OGG1. Electrophoresis mobility shift assays showed that CUT domains from CUX1, CUX2, and SATB1 stimulate the formation of a retarded complex between OGG1 and DNA containing 8-oxodeoxyguanine [10,11,39]. Binding of OGG1 to DNA was not observed with the corresponding control oligonucleotides containing a normal guanine base. Although none of the various oligonucleotides used in these assays contained a high-affinity binding site for CUT domain, previous studies indicated that the DNA-binding specificity of CUT domains can be somewhat relaxed [12,74]. Indeed, in some cases the CUT domains were able to produce a retarded complex of their own; however, in no case did we observe evidence of a stable ternary complex comprising OGG1 CUT domain and DNA [10,11,39]. Yet, because of the presence of a smear in some lanes, indicative of a transient interaction between CUT domains and DNA, we cannot exclude the possibility that transient binding of CUT domains to DNA helps OGG1 recognize DNA that contains an 8-oxoG [10,11,39]. Thus, there is a possibility that a ternary complex exists for a very short period of time. 

## 4. Stimulation of APE1 and Pol β Enzymatic Activities

Subsequent experiments with other enzymes of the base excision repair pathway indicated that CUT domains do not stimulate the enzymatic activity of other DNA glycosylases (Ramdzan, unpublished results). However, CUT domains were found in vitro to stimulate the function of the apurinic/apyrimidinic endonuclease 1, APE1 [61]. This finding raised the possibility that CUT domain proteins may contribute to the repair of bases other than oxidized purines. This hypothesis was confirmed by the observation that the resistance of glioblastoma cancer cells to treatment with temozolomide (TMZ), a mono-alkylating agent, was reduced by *CUX1* knockdown, but increased by ectopic expression of CUX1 [61]. These results were obtained both in MGMT-high and MGMT-low glioblastoma cell lines [61]. Importantly, ectopic expression of the CUT domains 1 and 2 (C1C2-NLS) protein was sufficient to increase resistance of glioblastoma cells to TMZ treatment and to combined treatment with TMZ and ionizing radiation, the latter representing the standard-of-care treatment for this type of cancer [61]. These results have significant implications considering that CUX1 is overexpressed in the majority of glioblastomas, as established from the immunohistochemical analysis of a panel of 150 glioblastomas with two different CUX1 antibodies [61]. These results confirmed partial results from The Cancer Genome Atlas (TCGA) research network and the Repository for Molecular Brain Neoplasia Data (REMBRANDT) showing overall reduced survival in glioblastoma patients with high and intermediate CUX1 expression levels [75,76,77]. In this context, it is important to stress that expression profiling studies provide only limited information on CUX1 since most oligonucleotides are derived from an exon that is unique to *CASP* (Cut alternatively spliced product), a messenger RNA coding for a protein that localizes to the Golgi [78,79]. Likewise, because the CASP-specific exons are located at the 3′-end, RNA-seq analysis often falsely interprets CASP RNA sequences as being specific to CUX1. 

**Figure 3:**
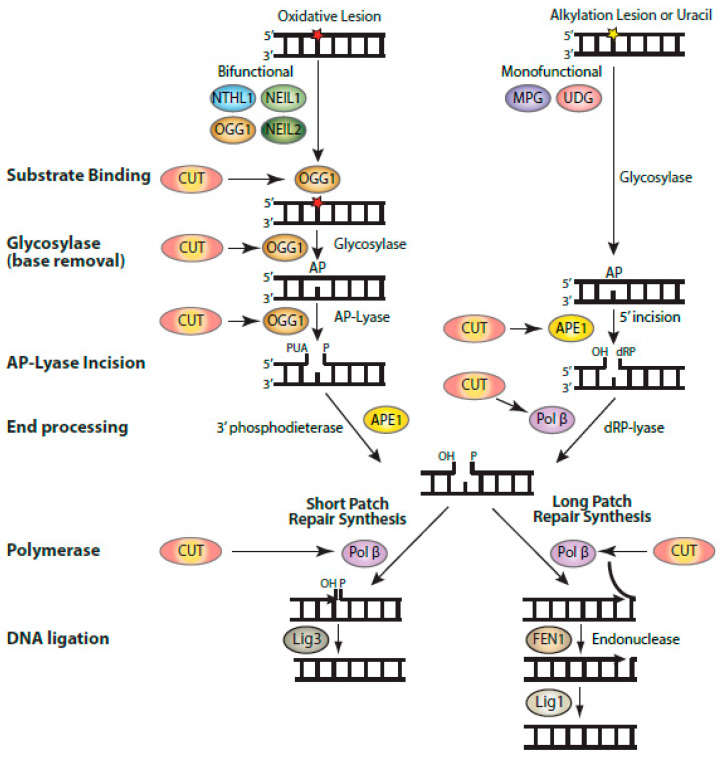
Biochemical activities of CUT domains in base excision repair. Diagrammatic representation of the steps involved in base excision repair, showing the enzymatic reactions that were found to be stimulated by CUT domains: substrate binding by OGG1, the glycosylase and AP-lyase activities of OGG1, the 5′-incision by APE1, and the polymerase and strand-displacement activities of DNA pol β. Base excision repair (BER) is initiated by a DNA glycosylase that is specific for the particular type of altered base [82]. DNA glycosylases for oxidized bases (OGG1, NTHL1, NEIL1, and NEIL2) carry out two enzymatic reactions: the glycosylase activity removes the oxidized base, while the AP-lyase activity introduces a single-strand break with a 3′-phospho-α,β-unsaturated aldehyde (3′-PUA) in the case of OGG1. This 3′-PUA is converted to a 3′-OH by the 3′-phosphodiesterase activity of APE1. UDG and MPG are monofunctional and only carry out the glycosylase activity, following which APE1 produces a single-strand incision with a 5′-deoxyribose phosphate (5′-dRP). This 5′-dRP is converted into a 5′-phosphate through the dRP-lyase activity of Pol β, which also introduces a single nucleotide in short-patch repair. It is not clear whether Pol β dRP-lyase activity or polymerase activity takes place first. When the 5′dRP is not removed, the pathway switches to long-patch repair, whereby a stretch of 2–13 nucleotides is synthesized by Pol β or Pol δ/ε aided by PCNA and RFC (not shown here). Not shown in this diagram is the XRCC1 scaffolding protein which interacts with Pol β and Lig3.

The increased resistance conferred by CUX1 or the C1C2-NLS protein to multiple genotoxic stresses such as H_2_O_2_, ionizing radiation, and temozolomide treatments could only partially be explained by the stimulation of OGG1 and APE1 enzymatic activities [9,39,61]. Indeed, the action of these enzymes produces single-strand breaks that can be more toxic than the original base damage if not repaired rapidly before replication [80,81]. This conundrum led us to investigate the effect of CUT domains on Pol β, the enzyme acting downstream of APE1 in the base excision repair pathway (reviewed in [82]). CUT domains were found in vitro to stimulate Pol β’ polymerase activities, in the context of both short-patch and long-patch repair, as well as the deoxyribose phosphate (dRP)-lyase activity [62]. The latter function, which converts a 5′-dRP group into a 5′-phosphate, is particularly important to enable ligation and, thus, completion of base excision repair (Figure 3) [83]. Indeed, when DNA ligase was added to a reaction containing Pol β and a substrate with a 5′-dRP at its single-strand break, CUT domains were able to stimulate DNA repair completion [62]. These results from in vitro assays received confirmation from the measurement of abasic sites in genomic DNA following treatment with TMZ. The number of abasic sites in genomic DNA was increased by *CUX1* knockdown, but decreased by ectopic expression of CUX1 [61,62]. In addition to the polymerase and dRP-lyase activities of Pol β, CUT domains were also shown in vitro to stimulate translesion synthesis by Pol β over intrastrand G-crosslinks [62]. These results were confirmed by observations made in cells; resistance to cisplatin, a chemotherapeutic drug that causes intrastrand crosslinks, was reduced by *CUX1* knockdown and was rescued by ectopic expression of the C1C2-NLS recombinant CUX1 protein [62].

## 5. Role of CUT Domain Proteins in the Resistance of Cancer Cells to Radiotherapy and Chemotherapy

Cancer cells in which the RAS pathway is activated produce elevated levels of reactive oxygen species (ROS) which cause oxidative DNA damage and, ultimately, cellular senescence (Figure 2) [19,20]. Cancer cells can adapt by increasing expression of ROS-scavenging proteins (reviewed in [23,35]) or by enhancing their capacity to repair oxidative DNA damage [9,10,11,38,61,62]. From gene expression analysis in DLD-1*^KRASG13D^*cells, increased DNA repair efficiency can be achieved though enhanced expression of BER enzymes such as APE1, PARP1, and Pol β, as well as BER accessory factors such as CUX1 [38]. The acute dependency of RAS-driven cancer cells on BER enzymes was made evident from the results of a genome-wide RNAi screen to identify synthetic lethal interactions with the *KRAS* oncogene. This screen identified four proteins involved in distinct steps of the BER pathway: NEIL2, XRCC1, POLβ, and LIG3 [37]. CUX1 was also in the list; however, at the time, its implication in DNA repair was not known [37]. We envision that cancer cells with enhanced DNA repair capability emerge via a process of natural selection. Following sustained activation of the RAS pathway, most cells in a population will be negatively affected by the excess in oxidative DNA damage, but a few rare cells that already express high levels of BER enzymes and accessory factors will continue to proliferate and will gradually represent an increasing fraction of the population. As a byproduct of this adaptation, RAS-driven cancer cells exhibit increased resistance to radiotherapy and a number of chemotherapeutic treatments [84,85,86,87,88]. Remarkably, ectopic expression of CUX1 was able by itself to confer increased resistance to various genotoxic treatments: H_2_O_2_ [9,39], ionizing radiation [38,61], temozolomide [61], combined treatment with radiation and temozolomide [61], and cisplatin [62]. These findings illustrate the phenomenon of co-option by cancer cells [89]. Enzymes and proteins, whose primary role is to maintain genomic integrity and prevent the emergence of mutant cells that could threaten the health of the organism, are now used by cancer cells to ensure their survival at the expense of the organism.

## 6. The Role of CUT Domain Proteins in Situations of Oxidative Stress

While the DNA repair function of CUT domain proteins (CUX1, CUX2, and SATB1) is required for the survival of cancer cells that exhibit high ROS levels, this biochemical activity does not appear to be essential to non-transformed cells in normal physiological situations. For example, *CUX1* knockdown caused a significant increase in DNA damage which was associated with a drastic decrease in cell proliferation in DLD-1*^KRASG13D^* and in Hs578T*^HRASG12D^*; however, no major adverse effects were observed in the matched control cell lines, DKO4 and Hs578Bst, which do not harbor a RAS oncogene [9]. Elsewhere, we observed that *CUX1* knockdown did not reduce the clonogenic efficiency of U251 glioblastoma cells and A549 lung cancer cells which exhibit low ROS levels [38]. Moreover, genetic inactivation of *CUX1* or *CUX2* in mouse does not cause embryonic lethality. In either case, mice are born, albeit with a number of developmental defects [90,91,92,93,94]. Furthermore, a striking phenotype was observed with mouse embryo fibroblasts (MEFs) from the *Cux1^−/−^* knockout mouse. In contrast to human diploid fibroblasts which gradually become senescent in culture over a period of 1 year because of telomere shortening, mouse cells have very long telomeres but MEFs senesce after 4 to 5 weeks when cultured in 20% oxygen because they accumulate oxidative DNA damage much more rapidly than human cells [95]. It should be noted that 20% oxygen represents a situation of oxidative stress, since the oxygen tension in the body is between 1% and 6% [96]. Indeed, wild type MEFs can proliferate indefinitely when maintained in 3% oxygen [95]. Likewise, *Cux1^−/−^* MEFs were able to proliferate well in a 3% oxygen atmosphere. However, when switched to a 20% oxygen incubator, *Cux1^−/−^* MEFs stopped proliferating immediately [39]. This proliferation block in 20% oxygen was linked to a defect in the repair of oxidative DNA damage [39]. The DNA repair defect and the capacity to proliferate in 20% oxygen could be rescued by ectopic expression of CUX1 or the shorter C1C2-NLS recombinant protein [39]. The absolute dependency on CUX1 function at 20% oxygen, but not at 3%, suggests that the role of CUX1 in base excision repair must be particularly important in situations of oxidative stress or in cell types that consume more oxygen. Neurons have very high rates of oxygen metabolism due to their dependence on aerobic oxidation of glucose as their source of energy [97]. Although the weight of the human brain represents only 2% of total body weight, it extracts approximately 50% of the oxygen and 10% of the glucose from the arterial blood [98,99]. Combined with the low level of antioxidant enzymes in the brain, elevated oxygen metabolism in neuronal cells is expected to produce significant ROS-induced oxidative DNA damage [100,101]. Interestingly, gene duplication during evolution led to the presence of two CUX genes in mammals: one that is expressed ubiquitously, CUX1, and one whose expression is restricted to neuronal cells, CUX2 [2,3]. Strikingly, *CUX2* knockdown in rat embryony cortical neurons caused a threefold increase in oxidative DNA damage [10]. It is tempting to speculate that duplication of the CUX gene during evolution may have been selected in part because of the protection conferred by CUX proteins against oxidative DNA damage in the brain.

## 7. Other DNA Binding Proteins Implicated in Base Excision Repair

It should be stressed that CUT domain proteins are not unique in having a direct role in base excision repair. YB-1 was reported to stimulate the enzymatic activities of NTH1 and NEIL2 [102,103,104], HMGB1 was reported to stimulate the functions of the APE1 and FEN1 endonucleases [105], and p53 was reported to stimulate the enzymatic activity of Pol β [106]. We consider that a systematic search for proteins that interact with BER enzymes would likely reveal many other transcription factors.

## Abbreviations

8-oxoG: 8-oxodeoxyguanines; APE1, apurinic/apyrimidinic endonuclease 1; BER, base excision repair; CUX, Cut homeobox; dRP, deoxyribose phosphate; GSK3-β, glycogen synthase kinase 3 β; HD, homeodomain; MEF, mouse embryo fibroblasts; MGMT, 6-*O*-methylguanine DNA methyltransferase; NLS, nuclear localization signal; OGG1, 8-oxodeoxyguanine DNA glycosylase; Pol β, polymerase β; SATB, special AT-rich sequence-binding protein; ROS, reactive oxygen species; RAS, rat sarcoma GTPase; SOS, son of sevenless.
